# A population‐scale multi‐omics blueprint of immune gene regulation

**DOI:** 10.1002/ctm2.70625

**Published:** 2026-02-11

**Authors:** Antonio Benedetto Ventura, Lucia Deligio, Giacomo Volpe

**Affiliations:** ^1^ Haematology and Cell Therapy Unit IRCCS Istituto Tumori “Giovanni Paolo II” Bari Italy; ^2^ Department of Pharmacy – Pharmaceutical Sciences University of Bari “Aldo Moro” Bari Italy

A central challenge in immunology and human genetics is the understanding of how genetic variation translates into immune diversity and disease susceptibility in a cell type‐specific manner.^1^ Although genome‐wide association studies (GWAS) have identified thousands of loci associated with immune‐mediated diseases, most variants lie in noncoding regions, obscuring their functional interpretation.^2^, ^3^ Bridging this gap requires comprehensive maps of gene regulation that integrate transcriptional output, chromatin accessibility, and genetic variation at single‐cell resolution.^4^ In this context, the Chinese Immune Multi‐Omics Atlas (CIMA) represents a landmark contribution, providing an unprecedented population‐scale, single‐cell multi‐omics resource for the human immune system.^5^

The most immediate novelty of CIMA lies in its **scale and integrative design**. By profiling more than **10 million peripheral blood mononuclear cells** from **428 healthy Chinese adults, covering an age span from 20 to 77 years,** using both single‐cell RNA sequencing (scRNA‐seq) and single‐cell ATAC sequencing (scATAC‐seq), the authors generate the largest immune single‐cell compendium to date. Importantly, this effort goes beyond descriptive cell atlases^6^, ^7^, ^8^ by integrating whole‐genome sequencing, plasma metabolomics, lipidomics and blood biochemistry, thereby creating a unified framework to study immune variation across molecular layers. Such breadth allows the authors to interrogate how genetic, epigenetic, transcriptional, and physiological variations intersect within the immune system.

A second major advance is the **depth of immune cell annotation**. Through iterative clustering and hierarchical classification, the study resolves **73 transcriptionally distinct immune cell types**, including rare and transitional populations that are often invisible in bulk or low‐resolution analyses. This refined cellular taxonomy provides the foundation for all downstream analyses and highlights the power of single‐cell approaches to capture immune heterogeneity across sex and age.^6^, ^9^, ^10^ The atlas reveals systematic age‐ and sex‐associated shifts in immune cell composition and molecular programs, reinforcing the notion that immune variation is both structured and context dependent.

One of the most impactful aspects of CIMA is the construction of **enhancer‐driven gene regulatory networks** for immune cells.^11^, ^12^ Using scATAC‐seq data, the authors identify more than **338,000 candidate cis‐regulatory elements**, most of which are distal and highly cell‐type specific. By integrating chromatin accessibility with gene expression, they link regulatory elements to target genes and transcription factors, resulting in GRNs that encode cell identities and functional states^13^ (Figure 1). These networks recover known lineage‐defining regulators while also identifying transcription factors active in rare or poorly characterised cell types. Importantly, the GRNs are not static: age‐associated and sex‐associated regulatory programs are resolved, revealing how immune regulation is remodelled across the human lifespan.^13^

**FIGURE 1:**
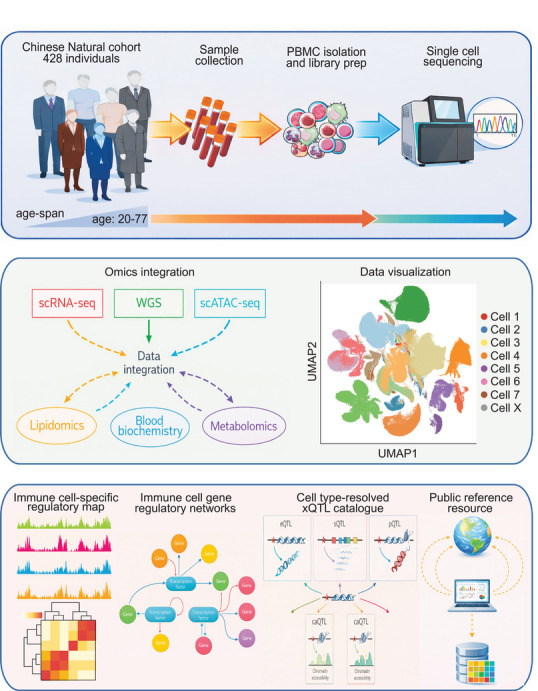
Overview of the single‐cell multi‐omics framework for immune regulatory analysis.

The integration of **genetic variation** elevates CIMA from an atlas to a mechanistic resource. By performing cell‐type‐resolved quantitative trait locus (xQTL) mapping, the authors identify **9600 expression quantitative trait loci** and **52,361 chromatin accessibility QTLs**. Strikingly, a large fraction of these associations is cell type specific, even when the regulated gene or regulatory element is active in multiple cell types (Figure 2). This observation underscores a key principle of immune genetics: the same genetic variant can have distinct regulatory consequences depending on the cellular context.^14^, ^15^, ^16^

**FIGURE 2:**
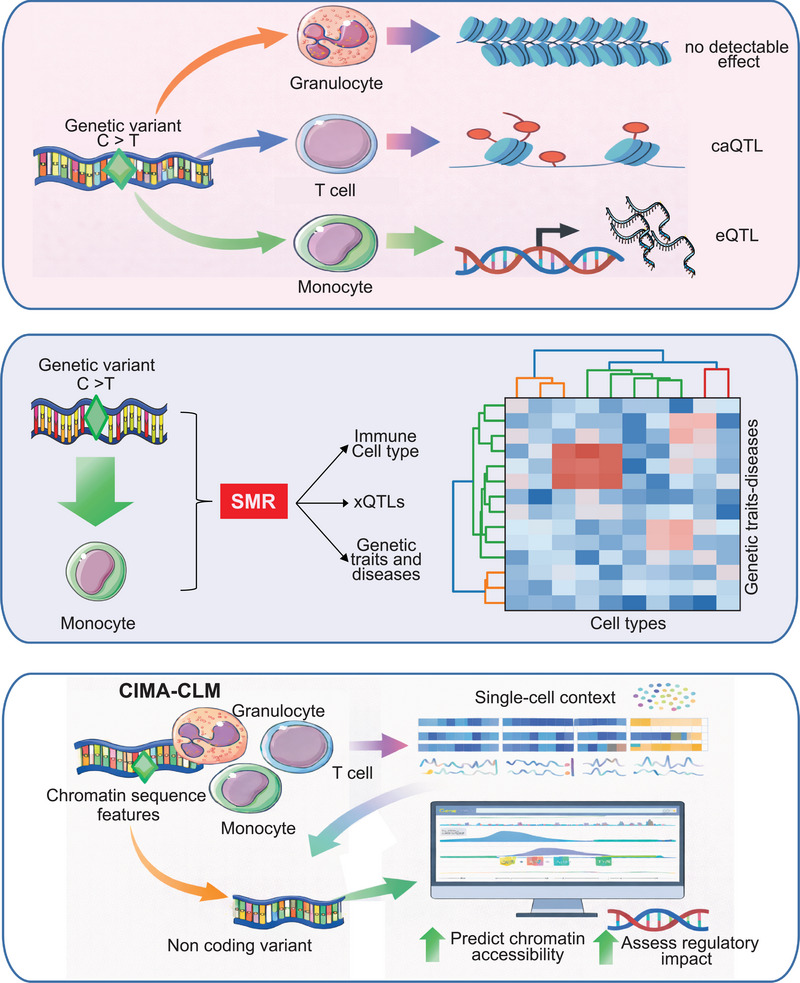
Cell type‐specific regulatory effects of genetic variants in immune cells.

Beyond static associations, the study captures **dynamic genetic effects** along differentiation trajectories in monocytes and B cells, demonstrating that genetic regulation itself can change during cellular maturation. This insight is particularly important for immune‐mediated diseases, many of which involve dysregulated differentiation or activation states rather than altered baseline cell identity.

A highlight of the work is the systematic integration of xQTL results with GWAS data using summary data‐based Mendelian randomisation.^17^, ^18^ This analysis uncovers more than **1000 pleiotropic associations** linking genetic variants to chromatin accessibility, gene expression, circulating inflammatory proteins, and disease risk, often in a **single immune cell type**. The example of a variant influencing asthma susceptibility through IKZF4 regulation specifically in regulatory T cells illustrates how CIMA enables precise mapping from genotype to disease‐relevant cellular mechanisms. Such cell‐type‐resolved interpretations are essential for translating genetic discoveries into therapeutic hypotheses.

Another innovative component of the study is the development of **CIMA‐CLM**, a cell‐type‐specific language model that integrates chromatin sequence features with single‐cell transcriptomic data to predict chromatin accessibility.^5^ This approach demonstrates strong concordance with experimental data and enables in silico mutagenesis to assess the effects of noncoding variants. By coupling experimental and computational frameworks, CIMA points toward a future in which regulatory variant function can be predicted and prioritised at scale.

The broader importance of CIMA extends beyond immunology. From a population genetics perspective, the atlas addresses a long‐standing bias toward European ancestry in functional genomics resources.^1^ The focus on a large Chinese cohort reveals regulatory variants that are rare or absent in other populations, emphasising the need for ancestry‐diverse datasets to fully understand human biology and disease. For the wider scientific community, CIMA provides a template for how population‐scale single‐cell atlases can be constructed and analysed at a comparable scale and resolution.

CIMA will also be invaluable as a **reference resource**. Its publicly accessible portal enables researchers to explore immune cell–specific regulatory landscapes, query xQTLs, and intersect their own data with a richly annotated atlas. Future studies of immune‐mediated diseases, vaccine responses, ageing and inflammation can leverage CIMA to interpret transcriptional or genetic signals in a precise cellular context. Moreover, the GRNs and regulatory annotations generated here offer a foundation for experimental validation and functional perturbation studies.

In summary, the CIMA represents a decisive step toward a mechanistic understanding of immune diversity. By uniting population‐scale genetics with single‐cell multi‐omics and advanced computational modelling, CIMA transforms how regulatory variation in the immune system can be studied. Its impact will be felt not only in immunology and human genetics but also across systems biology, precision medicine and computational genomics, serving as both a discovery engine and a lasting community resource.

Looking ahead, CIMA provides a foundation for extending immune regulatory maps to additional ancestries, disease states, longitudinal sampling and environmental perturbations. Integrating this framework with functional perturbation experiments, spatial profiling and clinical cohorts will enable causal dissection of immune regulation and accelerate the translation of genetic discoveries into precision immunology.

## AUTHOR CONTRIBUTIONS

Antonio Benedetto Ventura and Lucia Deligio wrote the manuscript and prepared the figures. Giacomo Volpe supervised the work and contributed to writing and editing the manuscript.

## CONFLICT OF INTEREST STATEMENT

The authors affiliated with the IRCCS Istituto Tumori “Giovanni Paolo II”, Bari, are responsible for the views expressed in this article, which do not necessarily represent the Institute. The authors declare no conflict of interest.

## ETHICS STATEMENT

This article is an editorial/commentary and does not report original research involving human participants or animals. Therefore, no ethical approval or informed consent was required.
